# Perception of one’s social environment and loneliness: results of the nationally representative “Old age in Germany (D80+)” study

**DOI:** 10.1007/s00127-024-02774-3

**Published:** 2024-10-01

**Authors:** André Hajek, Angelina Sutin, Martina Luchetti, Karl Peltzer, Nicola Veronese, Razak M. Gyasi, Pinar Soysal, Yannick Stephan, Antonio Terracciano, Hans-Helmut König

**Affiliations:** 1 Department of Health Economics and Health Services Research, University Medical Center Hamburg-Eppendorf, Hamburg Center for Health Economics, Martinistr. 52, 20246 Hamburg, Germany; 2 Florida State University College of Medicine, Tallahassee, FL USA; 3 Department of Health Education and Behavioral Sciences, Faculty of Public Health, Mahidol University, Bangkok, Thailand; 4 Department of Psychology, University of the Free State, Bloemfontein, South Africa; 5 Department of Psychology, College of Medical and Health Science, Asia University, Taichung, Taiwan; 6 Geriatrics Section, Department of Internal Medicine, University of Palermo, Palermo, Italy; 7 African Population and Health Research Center, Nairobi, Kenya; 8 Faculty of Health, National Centre for Naturopathic Medicine, Southern Cross University, Lismore, NSW Australia; 9 Department of Geriatric Medicine, Faculty of Medicine, Bezmialem Vakif University, Istanbul, Turkey; 10 Euromov, University of Montpellier, Montpellier, France

**Keywords:** Residential attachment, Neighborhood trust, Social isolation, Loneliness, Social exclusion, Social connectedness, Oldest old, Aged, 80 and above

## Abstract

**Objectives:**

To examine the association between perception of one’s social environment (in terms of residential attachment and neighborhood trust) and loneliness among the oldest old and whether these associations differ by living arrangement.

**Methods:**

We used data from the nationally representative “Old Age in Germany (D80+)” study that included individuals residing in private households and institutionalized settings. The analytic sample was 9,621 individuals (average age: 85.5 years, SD: 4.1 years; 62% female). Data collection took place from November 2020 to April 2021. Multiple linear regressions were conducted with adjustment for relevant covariates.

**Results:**

Higher residential attachment (β=-0.02, *p* < .05) and higher neighborhood trust (β=-0.12, *p* < .001) were associated with less loneliness. The latter association was moderated by living arrangement (β=-0.09, *p* = .04) such that the association between neighborhood trust and loneliness was stronger among individuals living in institutionalized settings compared to individuals in private households.

**Conclusion:**

Greater residential attachment and neighborhood trust, particularly among individuals living in institutionalized settings, are associated with less loneliness among the oldest old. Finding ways to improve perceived attachment and trust may assist in avoiding loneliness among older individuals.

## Introduction

A notable rise in the percentage of individuals aged 80 and above, commonly known as the “oldest old”, is anticipated over the next few decades [1]. A systematic review and meta-analysis found that just under 30% of the oldest old can be classified as “severely lonely” and a further 30% as “moderately lonely” [2]. Such higher loneliness can contribute to less satisfaction with life, unfavorable mental health, reduced perceived and actual longevity, and lower quality of life [3–7].

Because of its high prevalence and unfavorable correlates, there has been interest in the antecedents of loneliness among individuals aged 80 years and over (as an overview: [2]). Among individuals in this age group [8–10], for example, living alone and spousal loss is positively associated with loneliness. Less work has addressed the association between perceived neighborhood-related factors such as perception of one’s social environment and loneliness among the oldest old. There is, however, some evidence for these relations among older adult samples in general. For instance, one cross-sectional study found that higher levels of neighborhood social cohesion were associated with less loneliness among older adults in Hong-Kong in 2015–2017 before the COVID-19 pandemic (*n* = 1,037) [11]. Another study found that perceived neighborhood built environments (e.g., parks or green spaces) were associated with less loneliness in older adults in Europe before the pandemic [12]. A third study found favorable neighborhood factors (e.g., closeness of contact with neighbors; *n* = 6,688 observations) were associated with less loneliness among middle-aged and older individuals in Germany [13]. These studies thus provide evidence for the relation between neighborhood-related factors and loneliness.

There are several reasons why perception of one’s social environment may be associated with loneliness among the oldest old. In contrast to older people more generally (e.g., 60 years and older), oldest old individuals are more likely to have functional impairments [14] (e.g., dealing with financial issues or using the telephone) and are therefore more frequently accommodated in institutionalized forms of housing (importantly, however, most oldest old live independently in the community) [15]. In addition, some oldest old individuals may have lost friends and relatives and thus may be more reliant on their immediate surroundings for social interaction and support. Less attachment to where they live along with mistrust of their neighborhood may restrict access to social contact, particularly among individuals living in institutionalized settings (see also: [16]). Moreover, they may feel unsupported or overlooked when they need support. These factors may contribute to feelings of loneliness [13] and isolation [17].

In addition, for some, limited mobility and health problems can make it difficult to seek alternative sources of social contact, further increasing a sense of isolation, which may be a particular challenge among individuals living in institutionalized settings [16]. Feeling disconnected from the community may also increase loneliness, as older adults may feel overlooked or unsupported in times of need (see also: [18]). This issue is of great importance if, for example, oldest old individuals do not trust their neighbors and therefore do not take advantage of social interactions with people living most close to them. During the Covid-19 pandemic in particular, the oldest old individuals may have refused to seek help out of fear of being infected with the coronavirus [19] that could lead to further social withdrawal and promote loneliness [20]. A low sense of residential attachment could also be associated with a low quality of relationships in the place of residence (see also: [21]). For example, many contacts could be unidirectional in nature (e.g. with care workers) among the oldest old [22], which could have implications for the quality of the social relationships [21] that could lead to loneliness [23]. Furthermore, the oldest old individuals may long for friends and relatives who live further away [16], and those individuals may find it difficult to reach them, as many of oldest old no longer drive [24, 25]. Nursing home residents may find it difficult to see these friends and relatives again, especially during the pandemic, as there were contact restrictions in nursing homes to combat the pandemic.

Finally, similar to Akhter-Khan et al. [26] social relationship expectations framework (“respect: feeling valued and actively included”), on the micro level, the oldest old may yearn for recognition for their past contributions and included in decision-making as well as in daily activities in the neighborhood. On the meso-level, they may expect to be treated with respect, courtesy, and dignity as oldest old ones in their neighborhood. If these needs are not met, this can lead to loneliness [26].

The present study seeks to extend the literature on the relation between perception of one’s social environment and loneliness. The study focuses on the oldest old with data from a nationally representative sample of individuals aged 80 years and over, including both community-dwelling individuals and individuals residing in institutionalized settings. These associations may differ depending on the living arrangement whereby the relation between neighborhood-related factors and loneliness may be significantly more pronounced among individuals living in institutionalized settings compared to community-dwelling individuals. Overall, this knowledge is important to better understand loneliness and how the oldest old individuals feel in their neighborhood.

## Methods

### Sample

The “Old Age in Germany (D80+)” study is a large, nationally representative German sample of people aged 80 and over that included individuals living independently in the community and individuals living in inpatient facilities. The University of Cologne conducted the study in collaboration with the Cologne Center for Ethics, Rights, Economics, and Social Sciences of Health (ceres) and the German Center of Gerontology (DZA). The study was financially supported by the Federal Ministry for Family Affairs, Senior Citizens, Women and Youth (BMFSFJ).

Due to the pandemic, adjustments were made to the study design. Originally planned as face-to-face interviews, the study was switched to written questionnaires and supplemented by telephone interviews. The written questionnaires focused primarily on essential content, while the telephone interviews dealt with topics of somewhat lower priority. Detailed information about the methodology can be found elsewhere [27]. In total, over 10,000 people were surveyed (via written questionnaires), with data collection for the written questionnaire spanning from November 2020 to April 2021 [28].

The D80 + study obtained approval from the Ethics Committee of the Medical Faculty of the University of Cologne (the protocol number was as follows: 19-1387_1). Telephone interviews were conducted with the consent of the respondents. Respondents provided consent by completing and returning the questionnaire.

### Dependent variable: loneliness

Following previous research [2], an established single-item tool was used to measure loneliness ranging from 1 (“never or almost never”) to 4 (“always or almost always”), whereby higher values reflected more loneliness. The exact wording was: “How often have you felt lonely in the last week”.

Single item measures of loneliness are strongly associated with the UCLA loneliness scale [29], and such single-item tools to quantify loneliness have favorable psychometric characteristics [30, 31].

### Independent variables: residential attachment and neighborhood trust

Residential attachment was measured with the question “How closely do you feel connected to your living environment?” (1 = not at all; 2 = rather not; 3 = rather; 4 = very). Neighborhood trust was assessed with the question: “Can you trust the people in your neighborhood?” (1 = does not; 2 = rather not; 3 = applies; 4 = more likely; 5 = applies). “People in your neighborhood” was clarified with the following information: “neighbors outside the residential building, neighbors in the house, other residents or staff are to be included”. Similar items have been used to quantify such neighborhood-related factors [32–34].

### Covariates

Several covariates were included in the regression analysis based on previous research [35, 36]. Sociodemographic factors were gender (two categories: men; women), age (in years), marital status (five categories: married, living together with spouse; married but living separately from spouse; single; divorced; widowed), living arrangement (private living; institutionalized setting), and education (ISCED-2011 classification was used which has three categories: low, medium, or high education [37]). Lifestyle factors were social network size (continuous score) and the presence of sports activity (two categories: no or yes). Health-related factors were functional impairment and chronic illnesses. Functional impairment was measured with an adapted IADL tool developed by Lawton and Brody [38]. Participants were asked about seven tasks of daily living, including using the telephone, planning transportation, shopping, preparing meals, housework, taking medication and financial management. Each item was rated from 0 (needing help) to 2 (independent). Scores were averaged and reversed such that higher scores indicated greater functional impairment. The sum of up to 21 chronic conditions (in each case: 0 = absence, 1 = presence) was used to create a multimorbidity index [39, 40]: chronic conditions: myocardial infarction; heart failure; hypertension; stroke; mental illness; cancer; diabetes, respiratory or lung disease; back pain; gastrointestinal disease; kidney disease; liver disease; blood disease; joint or bone disease; bladder disease; sleep disorders; eye disease or visual impairment; ear disease or hearing impairment; neurological disease; (blood) vascular disease; thyroid disease.

### Statistical analysis

Descriptive statistics were used to describe the analytic sample. Multiple linear regressions (with cluster-robust standard errors) were used to examine the association of perception of one’s social environment with loneliness. Unadjusted and adjusted regressions were conducted. Variance inflation factors (VIFs) were calculated: The mean VIF was 1.34, and the highest VIF was 1.95, indicating no multicollinearity issues. To explore whether the association between neighborhood-related factors and loneliness differed depending on living arrangement, corresponding interaction terms were included in the regression model.

Non-response and the disproportionate sampling design such as oversampling of men was addressed using sampling weights [27]. Listwise deletion was used to address missing values. In our main model, a full information maximum likelihood (FIML) strategy [41] was employed to address missing values. Statistical significance was established at *p* < .05 in this study. Statistical analyses were performed using Stata 18.0 (Stata Corp., College Station, Texas).

## Results

### Sample characteristics

The weighted analytic sample is in Table 1. Average age was 85.5 years (SD: 4.1, range 80 to 100 years) and 61.6% of participants were women. The average level of residential attachment was 2.8 (SD: 1.0) and average neighborhood trust was 4.1 (SD: 1.1). The average loneliness level was 1.7 (SD: 0.8).

**Table 1:** Sample characteristics of the analytical sample (*n* = 9,621 individuals, weighted)

*Variables*	*Mean (SD) / n (%)*
Residential attachment (from 1 to 4, whereby higher values correspond to higher residential attachment)	2.8 (1.0)
Neighborhood trust (from 1 to 5, whereby higher values correspond to higher neighborhood trust)	4.1 (1.1)
Loneliness (1 to 4, whereby higher values correspond to higher loneliness levels)	1.7 (0.8)
Gender	
Men	3698 (38.4%)
Women	5923 (61.6%)
Age	85.5 (4.1)
Marital status	
Married, living together with spouse	3860 (40.6%)
Married, living separated from spouse	98 (1.0%)
Divorced	454 (4.8%)
Widowed	4694 (49.4%)
Single	403 (4.2%)
Living arrangement	
Private household	8682 (90.2%)
Institutionalized setting	939 (9.8%)
Education	
Low education	2122 (22.8%)
Medium education	4804 (51.6%)
High education	2377 (25.5%)
Sports activity	
No	4162 (43.3%)
Yes	5459 (56.7%)
Social network size	7.7 (6.6)
Chronic conditions (count score, 0 to 21, whereby higher values correspond to a higher number of chronic conditions)	4.7 (2.7)
Functional impairment (0 to 2, whereby higher values correspond to a higher functional impairment)	0.6 (0.7)

## Regression analysis

The results of the unadjusted and adjusted linear regressions are in Table 2. According to the unadjusted regressions, higher residential attachment (β=-0.02, *p* < .05) and higher neighborhood trust (β=-0.12, *p* < .001) were significantly associated with less loneliness in the unadjusted models. The results based on listwise deletion for missingness (second column) were nearly identical to results based on FIML for missingness (third column). The associations remained significant in the fully-adjusted model for both higher residential attachment (β=-0.02, *p* < .05) and higher neighborhood trust (β=-0.06, *p* < .001), although the association with residential attachment was borderline significant (*p* < .10) controlling for sociodemographic factors and/or lifestyle factors (Table 2).

**Table 2:** Association between neighborhood-related factors and loneliness. Results of linear regressions (unadjusted and adjusted)

Independent variables	Loneliness	Loneliness	Loneliness	Loneliness	Loneliness	Loneliness	Loneliness	Loneliness
	With listwise deletion to address missings				With FIML to address missings			
Residential attachment	-0.02*	-0.02+	-0.02+	-0.02*	-0.02*	-0.02+	-0.02+	-0.02*
	(-0.04 to -0.002)	(-0.03 to 0.00)	(-0.03 to 0.00)	(-0.04 to -0.00)	(-0.04 to -0.003)	(-0.03 to 0.00)	(-0.04 to 0.00)	(-0.04 to -0.01)
Neighborhood trust	-0.12***	-0.08***	-0.07***	-0.06***	-0.12***	-0.08***	-0.07***	-0.06***
	(-0.14 to -0.10)	(-0.10 to -0.06)	(-0.09 to -0.06)	(-0.07 to -0.04)	(-0.14 to -0.10)	(-0.10 to -0.06)	(-0.09 to -0.06)	(-0.07 to -0.04)
Sociodemographic covariates		✓	✓	✓		✓	✓	✓
Lifestyle-related covariates			✓	✓			✓	✓
Health-related covariates				✓				✓
Observations	9,831	9,393	9,198	9,024	9,831	9,831	9,621	9,621
R²	0.03	0.18	0.18	0.23	0.001	0.18	0.18	0.22

*** *p* < .001, ** *p* < .01, * *p* < .05, + *p* < .10; unstandardized beta-coefficients are displayed; 95% CI in parentheses; cluster-robust standard errors were calculated (using the primary sampling unit); sampling weights were applied; furthermore, adjustment was made for sample cells; Sociodemographic covariates: gender, age, marital status, living arrangement, and education; Lifestyle-related covariates: sports activity, and social network size; Health-related covariates: chronic illnesses and functional impairment

We tested whether these associations differed by living arrangement. The interaction term for living arrangement x neighborhood trust was significant (β=-0.09, *p* = .04), which indicated that the association between neighborhood trust and loneliness was stronger among individuals living in institutionalized settings (β=-0.10, *p* = .03) compared to individuals living in community settings (β=0.05, *p* < .001). The interaction term between living arrangement and residential attachment was not significant (β = 0.02, *p* = .72).

## Discussion

The aim of this study was to examine the association between residential attachment and neighborhood trust and loneliness among the oldest old individuals based on large, nationally representative data from Germany. A second aim was to examine whether these associations differ depending on the living arrangement. Higher residential attachment and neighborhood trust were both associated with less loneliness among individuals aged 80 years and over. The association between neighborhood trust and loneliness was moderated by living arrangement such that the association was more pronounced among individuals living in institutionalized settings. This study, which focused *on the oldest old* during the pandemic, extends current knowledge on the association between perceived neighborhood-related factors and loneliness, which was based mainly on studies of middle-aged or older adults (e.g [11–13]).

A somewhat related study by Gibney et al. [42] showed that age-friendly urban environments were associated with lower levels of loneliness among adults aged 55 years and over in four Irish cities. Another study showed that chatting with neighbors was associated with less loneliness among individuals aged 65 to 79 years and among individuals aged 80 years and over based on data from the Shanghai Urban Survey in 2016 and 2017 [43]. Of note, a review (not specifically focusing on the oldest old) [44] emphasized the relevance of subjective assessments of neighborhood-related factors for loneliness. The relevance of neighborhood-related factors (such as contacts) for loneliness specifically among older adults has also been demonstrated by a past review and meta-analysis [23]. Our present study builds upon this knowledge by showing an association between neighborhood-related factors and loneliness among the oldest old individuals in Germany.

Given the relatively higher rate of functional impairment and loss of friends and relatives among the oldest old individuals, the association between residential attachment and loneliness is reasonable. Regarding potential mechanisms, with fewer friends and relatives nearby, oldest old individuals may heavily rely on immediate surroundings for social connection. Thus, neighbors can become particularly important due to their proximity. When attachment to their living environment is insufficient, individuals may feel lonely [23]. Moreover, low neighborhood trust combined with fears of Covid-19 infection may further isolate oldest old individuals (particularly in institutionalized settings) that may hinder access to required support [16]. Additionally, unidirectional relationships - such as those with care workers - may lack depth [21]. This may affect the quality of social relationships which may be particularly important for nursing home residents who may yearn for distant relatives and friends [16]. In line with the social relationship expectations framework, a lack of appreciation may also reduce the quality of the relationship and lead to loneliness [26].

Our study has both strengths and limitations. A key strength is that data came from a very large sample which is generalizable to individuals aged 80 years and over living in Germany. Moreover, individuals residing in institutionalized setting as well as community-dwelling individuals were covered in the D80 + study. We applied sampling weights and missing data were addressed using a FIML approach. Limitations particularly refer to the cross-sectional design of the D80 + study. The item to quantify loneliness has a high face validity, has performed well in previous research, and is closely connected to multi-item scales [29]; however, it may not fully capture the complexity of the loneliness phenomenon (covering social and emotional loneliness). The single-item assessments of attachment and trust have similar limitations [45, 46]. Thus, we recommend future research with multi-item scales to confirm our present findings.

## Conclusion and future research

Our study showed an association of both higher residential attachment and neighborhood trust (particularly among individuals living in institutionalized settings) with lower loneliness among the oldest old individuals. Efforts to improve these neighborhood-related factors may assist in avoiding loneliness among individuals aged 80 years and over. Upcoming studies could also explore whether the association between these neighborhood-related factors and loneliness differs between countries (e.g., individualistic vs. collectivistic cultures). Moreover, future longitudinal studies could also investigate how changes in neighborhood-related factors can contribute to loneliness in the long run.

## Data Availability

All data are available from the German Centre of Gerontology. For further details (application for data use): https://www.dza.de/en/research/fdz/access-to-data/application.
